# Sweat components as a promising monitoring tool for systemic diseases

**DOI:** 10.1016/j.jphyss.2026.100068

**Published:** 2026-03-15

**Authors:** Changqi Shi, Ziyi Chen, LuLu Zhang, Xiaomeng Zhang, Xiaoyu Liang, Lan Yan, Xiaopeng Hao, Guoju Dong, Cheng Lu, Luqi Huang

**Affiliations:** a Institute Of Basic Research In Clinical Medicine,China Academy Of Chinese Medical Sciences, Beijing 100700, China; b National Resource Center for Chinese Materia Medica, China Academy of Chinese Medical Sciences, Beijing, 100700, China; c Xiyuan Hospital, China Academy of Chinese Medical Sciences, Beijing, 100091, China

**Keywords:** Sweat, Sweat components, Monitoring tool, Dermatological afflictions, Metabolic diseases

## Abstract

Sweat is a complex biological fluid primarily responsible for thermoregulation, containing diverse organic and inorganic components derived from plasma and interstitial fluids. Its secretion is tightly regulated by hypothalamic–sympathetic pathways and reflects systemic physiological status. Recent studies reveal that sweat composition dynamically changes with various systemic diseases, providing diagnostic, mechanistic, and prognostic insights. Abnormalities in sweat components, such as electrolytes, glucose, lactate, amino acids, and proteins, mirror underlying metabolic, endocrine, immune, and neural dysregulation. This review synthesizes current evidence on how these alterations arise from specific physiological mechanisms—including transdermal diffusion, transporter-mediated secretion (e.g., GLUT2, CFTR), ductal reabsorption, and autonomic control—linking sweat gland biology to systemic homeostasis. These insights support the clinical development of sweat as a non-invasive biofluid for disease monitoring.

## Introduction

Sweat is essential for thermoregulation and reflects systemic physiological status. and is produced primarily by plasma and interstitial fluid through richly vascularized sweat glands [1], [2]. Sweat is generally weakly acidic (pH 4.0--6.8) and is composed mainly of water (99%), organic compounds, and inorganic substances [3]. The acidic environment of sweat provides a natural barrier for the skin, and the water, urea, and lactic acid in sweat act as natural moisturizers to prevent skin dryness and maintain barrier function [4].

Sweat secretion is mainly regulated by the preoptic area of the hypothalamus. This area integrates temperature sensory input signals and activates the sympathetic cholinergic fibers that innervate the sweat glands, thereby establishing a core neurophysiological pathway [5]. Additionally, Benzinger considered the correlation between body temperature and the sweating rate and suggested that, in a healthy state, heat production and heat dissipation in the body are in dynamic balance [6]. In addition to thermoregulation, sweat serves as an important "window" into overall physiological conditions. Its composition of ions, metabolites, and proteome undergoes dynamic changes due to factors such as inflammation, endocrine disorders, and neurological dysfunction [7], [8], [9]. These alterations arise not arbitrarily, but through defined physiological processes—including transdermal diffusion, active transport via glandular transporters (e.g., GLUT2, CFTR), and neural or hormonal modulation of secretion [10]. Therefore, similar to blood, urine, and saliva, sweat can also be used for the clinical analysis of various metabolic or nonmetabolic diseases. Moreover, many diseases, such as cholinergic urticaria and cancer, result in abnormal sweat secretion, which has prompted corresponding treatment research. Sweat is thus gaining increasing recognition as a valuable, non-invasive diagnostic fluid for monitoring metabolism, inflammation, and other systemic diseases [11], [12].

## Biological basis of sweat

### Anatomy and physiology of the sweat glands

**Fig. 1:**
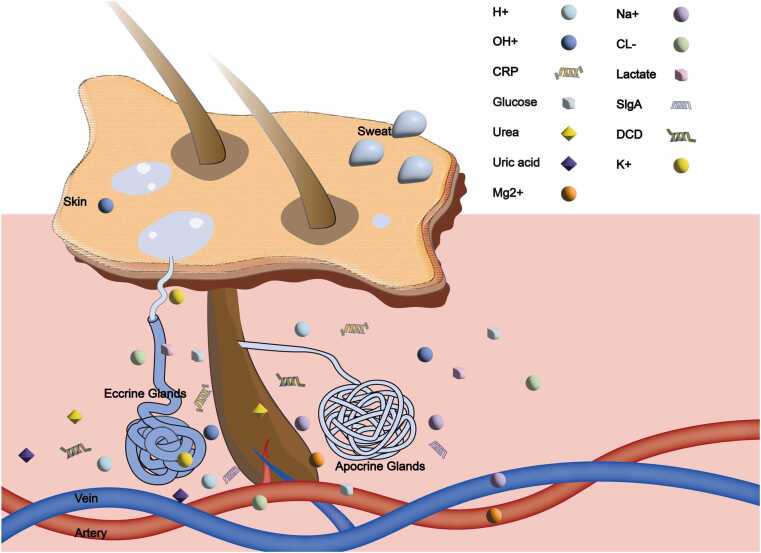
| **Sweat gland structure and physiology.** Human sweat glands are categorized into two main types: eccrine and apocrine. Eccrine glands, which are distributed throughout the body, consist of a nonbranching duct and a secretory coil, which includes clear cells, dark cells, and myoepithelial cells. These components play crucial roles in thermoregulation and the secretion of a water-rich, transparent fluid.

The human body is equipped with approximately 2–5 million sweat glands, which are mainly divided into two categories: eccrine and apocrine glands [1], [13] (Fig. 1). Human sweat secretion is primarily regulated by the central nervous system through the sympathetic pathway. In sweat glands, acetylcholine (ACh) is released by sympathetic preganglionic neurons. These neurons mainly originate from the T3–L2 spinal cord segments, and the sweat glands in different body regions are regulated by the corresponding segments. Eccrine glands are extensively distributed throughout the body, and their structure includes ducts and secretory coils, which are composed of clear cells, dark cells, and myoepithelial cells [14], [15], [16]. These cells work together to regulate body temperature and secrete a clear liquid that is rich in water [1], [17]. In contrast, apocrine glands are more developed during puberty because of the influence of sex hormones, and their secretions are more viscous and are primarily concentrated in specific areas, such as the armpits [14]. Notably, there is a type of sweat gland called the apoeccrine gland, which has characteristics of both eccrine and apocrine glands, but the existence of these glands is still a matter of debate in the scientific community [18], [19].

The varying ratios of eccrine and apocrine glands across body regions, combined with microbial activity, result in significant compositional differences in sweat [14]. The secretions of apocrine glands are rich in lipids, whereas eccrine glands contain a wide variety of trace nutrients [15], [16], [20]. In addition, the secretions of sebaceous glands influence the final composition of sweat to a certain extent [2]. The basic components of sweat mainly include water and sodium chloride (NaCl), as well as various other components, such as trace nutrients and metabolites [15], [20]. The components and concentrations of some sweat are shown in Table 1. Among them, metabolites such as glucose and lactate reflect the body's metabolic status, while ions like Na+ and K+ play a vital role in maintaining internal homeostasis [21], [22], [23], [24]. Additionally, amino acids, inflammatory cytokines, and various proteins also perform essential physiological functions in the body [25], [26], [27], [28], [29], [30]. These components are closely linked to the onset and progression of numerous diseases.

**Table 1:** Physiological components and concentrations of sweat.

Biomarkers		Concentration	Ref.
Metabolic products	Glucose	0.02–0.6 mM	[21]
	Lactate	16–30 mM	[22]
	Urea	4–12 mM	[15]
	Uric acid	2–10 mM	[31]
	Cortisol	8–140 μg·L−1	[32]
Ions	CL-	10–100 mM	[15]
	Na+	10–90 mM	[23]
	K+	1–16 mM	[24]
	Ca2+	0.41–12.4 mM	[31]
	Mg2+	0.02–0.4 mM	[23]
	Zn2+	0.01–0.002 mM	[23]
	Cu2+	100–1000 μg·L^−1^	[31]
	PH	5.5–6.5	[33]
Amino acid	Pyrrolidone carboxylic acid, urocanic acid, serine, histadine, ornithine, glycine, alanine, aspartic acid, lysine	-	[25], [26]
Inflammatory factor	IL1α、IL-1β、IL-6、TNF-α、IL-8、TGF--β	0.07–1 pM	[27]
Proteins	DCD、sIgA、IgE、CRP		[28], [29], [30]

### Functions of sweat

Sweat glands are versatile organs that serve both exocrine and excretory functions [34]. They play pivotal roles in the body's intricate systems, regulating temperature, participating in metabolism, and bolstering host defense mechanisms [2], [14], [16], [35]. During exercise, heat production by muscles causes an increase in body temperature, activating this mechanism and prompting sweat glands to secrete sweat. The evaporation of sweat (each gram of sweat absorbs 580 calories of heat) effectively maintains the core body temperature within a safe range of 37°C ± 1°C. The heat - dissipation efficiency can reach 10 times that of the resting state during high - intensity exercise [36]. In addition to its coiled, network-like structure that continues into a straight duct opening at the epidermal surface, the sweat gland secretes fluid into the duct, where much of the Na⁺, Cl⁻ and water is reabsorbed before final discharge onto the skin. Because this exocrine fluid also contains urea and other small solutes, the gland is functionally regarded as a “third kidney” [37]. The total amount of urea excreted in sweat accounts for about 0.1–1% of the amount excreted by the kidneys. This indicates that there is a physiological continuity between sweat gland excretion and kidney clearance. Especially in patients with renal failure, the urea content in sweat far exceeds that in serum. Therefore, sweat can serve as an important indicator in the diagnosis and treatment of diseases [38]. Although sweat glands respond to aldosterone by increasing Na⁺ reabsorption in the duct, thereby reducing Na⁺ concentration in sweat, this response exhibits a much longer time lag compared to the kidneys, and thus its physiological significance is considered debatable [23].

Research has revealed the involvement of the aquaporin-5 (AQP5) protein in sweat, facilitating the transmembrane transport of water molecules [6], [39]. Furthermore, acid—base balance plays a crucial role in maintaining osmotic pressure and body fluid volume [40]. The skin, an effective biochemical fortress, provides a robust defense system [3], [40]. The water—oil film, a blend of sweat and sebum, forms a formidable first line of defense [40]. Dermcidin(DCD), an antimicrobial peptide present in sweat, is an indispensable component of the skin's defensive arsenal [41], [42], [43]. Additionally, sweat contains various inflammatory factors that stimulate immune cells, triggering an inflammatory response and further enhancing the resistance of the skin to external stimuli [28], [29], [44]. In essence, sweat glands are multifaceted guardians of our physiological equilibrium.

## The potential of sweat as a diagnostic tool

Human body fluids include interstitial fluid, saliva, blood, urine, sweat, and others. Non-invasive human fluid sampling typically involves tear fluid, interstitial fluid (ISF), exhaled breath condensate, saliva, wound exudate, and sweat, among others [31]. Tear fluid secreted by the lacrimal glands contains abundant salts, enzymes, proteins, and other components, and is commonly used for diagnosing eye diseases [45], [46]. Similarly, saliva secreted by salivary glands and ISF that diffuses out from capillaries are also widely employed in monitoring a range of diseases due to their rich content of biomarkers [47], [48]. As an accessory organ of the skin—the body's largest secretory organ, sweat glands are distributed almost throughout the entire body [49]. Their easy accessibility, low sample volume requirement for analysis, and rich composition make them a leading choice for non-invasive monitoring [33]. The physiological mechanisms underlying these changes are detailed in Table 2, while disease-specific alterations in sweat components (including pH) are summarized in Table 3 [50].

**Table 2:** Pathophysiological mechanisms underlying abnormal changes in sweat components.

Sweat component	Normal Physiological Role	Pathophysiological Mechanism of Abnormal Change	Ref.
Glucose	Minor excretion via sweat (low baseline)	Elevated blood glucose increases the concentration gradient across the skin, promoting passive diffusion into sweat. Upregulation of GLUT2 transporters in eccrine glands may further enhance transdermal glucose movement.	[57], [130]
Lactate	Low physiological levels (anaerobic metabolism byproduct)	Systemic lactate accumulation—due to impaired tissue perfusion or mitochondrial dysfunction in conditions like heart failure or diabetes—leads to increased diffusion of lactate into sweat.	[64], [157]
Uric acid	Minor renal excretion (urine primary route)	Reduced renal excretion in hyperuricemia or uremia causes systemic buildup of uric acid, which then passively diffuses into sweat down its concentration gradient.	[65], [81], [91]
Urea	Minor excretion via sweat (healthy kidneys handle urea)	In uremia, markedly elevated plasma urea creates a strong driving force for passive diffusion into sweat. Concurrently, reduced sweating limits dilution, leading to high concentrations and possible crystallization on the skin (“uremic frost”).	[37], [62], [97]
Cl⁻	Regulates sweat osmolarity (reabsorbed in duct)	In cystic fibrosis, loss-of-function mutations in CFTR impair chloride reabsorption, resulting in high sweat chloride levels. In hyperthyroidism, increased sweat production may dilute chloride concentration despite normal reabsorption.	[2], [85]
K⁺	Near serum concentration (stable baseline)	In uremia, severe hyperkalemia combined with reduced sweat flow overwhelms ductal reabsorptive capacity, leading to markedly elevated sweat K⁺ (over four times plasma levels). In mild AD, enhanced ductal reabsorption may lower sweat K⁺.	[29], [85], [99]
Cortisol	Stress hormone (low in sweat)	Cortisol likely enters sweat via passive diffusion from plasma, with no known active transport mechanism. Sweat concentrations thus correlate with hypothalamic–pituitary–adrenal (HPA) axis activity.	[49], [76], [109], [119], [149], [150], [152]
25-(OH)₂-D₃	Regulates calcium signaling in eccrine glands	Higher serum levels of 25-(OH)D₃ are associated with improved sudomotor function, possibly through enhanced calcium-dependent signaling and maintenance of nerve integrity in sweat glands.	[26], [56]
Neuropeptide Y (NPY)	Stress-related signaling in sweat glands	Elevated stress leads to increased plasma NPY, creating a concentration gradient that promotes diffusion into sweat, which correlates with anxiety-induced sweating.	[55], [112]

**Table 3:** Disease-specific alterations in sweat component concentrations (partial diseases).

Disease	Sweat Glucose	Sweat Lactate	Sweat Urea	Sweat Uric acid	Sweat Cl⁻	Sweat K⁺	Sweat pH	Sweat Cortisol
Diabetes mellitus	↑	↑	↑	-	↑	↑	-	-
Hyperuricemia/Gout	-	-	-	↑	-	-	↓	-
Hyperthyroidism	-	-	-	↑	↓	↑	-	-
Hypothyroidism	-	-	-	↓	↑	↓	-	-
Uremia	-	-	↑	-	-	↑	-	-
Cystic Fibrosis	-	-	-	-	↑	-	↓	↓
Heart Failure	-	↑	-	-	↓	-	↑	↓
Atopic Dermatitis	↑	↓	↓	-	-	↓	↑	-

### Endocrine and metabolic diseases

**Endocrine and metabolic diseases** refer to a category of diseases caused by abnormalities in the body's metabolic processes. On the basis of the type of metabolic disorder and the systems affected, endocrine and metabolic diseases can be divided into several types, which are generally classified as follows:

#### Carbohydrate metabolism abnormalities

##### Diabetes mellitus (DM)

DM is a metabolic disease characterized by a disorder in carbohydrate metabolism, where glucose cannot be fully utilized, leading to elevated blood sugar levels [51]. Type 1 diabetes Mellitus (T1DM) is generally caused by the autoimmune destruction of pancreatic beta cells, resulting in an obstacle for the pancreas to synthesize and secrete insulin Type 2 diabetes mellitus (T2DM) on the other hand, is the result of a combination of insulin resistance and insufficient secretion [51]. According to statistics, the global prevalence of DM for individuals aged 20--70 years was 10.5% in 2021, and it is projected to rise to 783.2 million by 2045 [52]. In 2021, 6.7 million people worldwide died from DM and related causes. There is a direct relationship between the blood glucose concentration and the risk of complications in the kidneys, retina, and nervous system [51], [52]. Therefore, screening and diagnosis of DM are particularly important. The current methods for the diagnosis and detection of DM are relatively comprehensive, but the use of multiple blood samples is painful for patients, making noninvasive detection of disease highly popular.

**Fig. 2:**
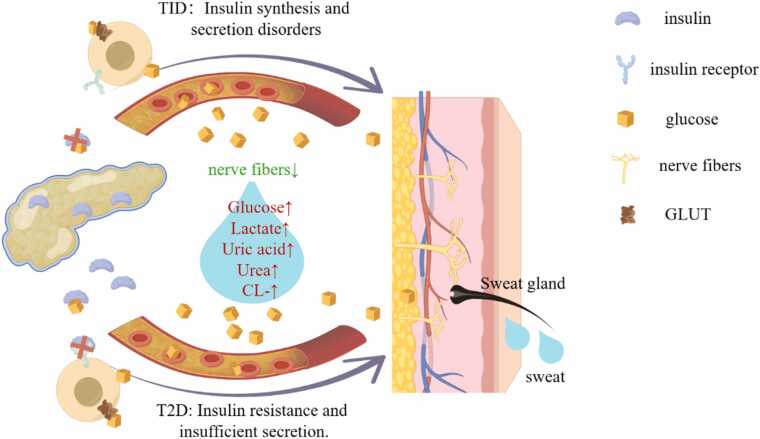
**Concentrations of various components in the sweat of diabetic patients.** DM is characterized primarily by elevated blood glucose levels. The composition of sweat in diabetic individuals is significantly different from that in healthy individuals, with markedly increased concentrations of glucose, lactate, and chloride ions. The levels of urea and uric acid also change. These alterations could serve as potential biomarkers for the diagnosis and monitoring of DM.

Diabetic patients often exhibit abnormal sweating function. The core mechanism is closely related to the impairment of sweat gland innervation caused by long - term hyperglycemia - induced autonomic neuropathy (AN). Once the autonomic neuropathy affects the sympathetic nerves that innervate the sweat glands, it will lead to progressive damage to their structure and function. In the diabetic state, in addition to the impairment of sympathetic cholinergic innervation detailed above, the fluctuations in the levels of circulating catecholamines (NA, ADR) and the changes in the reactivity of their receptors have become important DM - related factors affecting sweat gland function. Diabetic patients are prone to hypoglycemic events. Hypoglycemia strongly triggers the sympathetic - adrenal system to release large amounts of NA and ADR [14]. Additionally, metabolic disturbances caused by hyperglycemia directly damage nerve axons and Schwann cells, leading to demyelination and axonal degeneration [53]. This ultimately manifests as a reduction in sweat gland nerve fiber density (SGNFD) and impaired sweat gland function.After evaluating 36 diabetic patients and 72 healthy subjects, Gibbons et al. found that the sweat gland innervating nerves in diabetic patients were significantly reduced compared with the control group. It was found that as the neuropathy worsened, the SGNFD in the distal calf gradually decreased (r = -0.81) [54]. Subsequent quantification of sweat gland nerve fibers revealed that sweat gland nerve fiber density (SGNFD) can be used to distinguish between mild diabetic neuropathy patients and healthy controls [55]. Vinni Faber Rasmussen et al. reported that the length of hair follicle nerve fibers and the average value of nerve fibers in adolescents with T1D were significantly reduced and that the sweat response was decreased or absent [18]. Moreover, SGNFD depends on the duration and level of DM (glycated hemoglobin greater than 8.5%). Diabetic neuropathy-induced gustatory sweating is accompanied by abnormal sweating in the face and upper trunk [16]. In Goodman's report, anhidrosis can be detected in 29% of patients, which is precisely the comprehensive manifestation of the above-mentioned multi-level damages (nerve loss, receptor dysfunction, glandular atrophy) in patients with diabetic autonomic neuropathy (AN) [16]. Chen tested the 25-(OH)₂-D₃ levels and sweating function in 1021 patients with T2DM. Through multiple regression analysis, it was found that serum 25-(OH)₂-D₃ was positively correlated with sweating function, providing a potential intervention target for DM management [56] (Fig. 2).

Glucose: Glucose is one of the main metabolic products secreted in sweat, and its concentration in sweat is much lower than that in the blood. Specifically, the blood glucose concentration ranges from 2 to 40 mM, whereas the glucose concentration in sweat is only 0.02–0.6 mM [21]. For patients with DM, the glucose content in sweat ranges from 0.28 to 1.11 mM, which is much higher than that in the normal population [57]. This elevation is attributed to hyperglycemia-driven passive diffusion across the secretory coil epithelium and upregulated expression of GLUT2 transporters, which enhance glucose flux into sweat [58]. Consistent with this mechanism, a strong positive correlation between sweat and blood glucose is observed in diabetic patients, whereas no such relationship exists in normoglycemic individuals, suggesting that sweat glucose becomes sensitive to systemic glycemia only when plasma concentrations exceed physiological thresholds. Due to its low abundance, sweat glucose measurement typically requires pretreatment to remove contaminants [7], [59].

Lactate (LA): LA, a product of glycolysis and anaerobic glucose metabolism, is known as a marker of hypoxia and is also one of the main metabolites that can be detected in sweat. The lactate content in the sweat of healthy individuals varies within the range of 16–30 mM and can exceed 50 mM under conditions of extreme intensity training and stress testing [22]. Jin et al. measured the value of DL-LA in sweat from the elbow via a novel mass spectrometry probe-labeled dry sweat spot paper (DSSP) method and reported a medium positive linear correlation between the D/L-LA ratio in human sweat and fasting blood glucose levels, suggesting that the D/L-LA ratio in human sweat can be used as a potential biomarker for DM screening [60], [61].

Ions: The concentration of ions such as Na^+^, K^+^, and Cl^-^ in sweat also changes with the progression of the disease. The concentration of chloride ions in the sweat of patients with DM is 1.8 times greater than that in the sweat of normal individuals [37]. Sweat electrolyte conductivity (ESC) detection, as an objective and sensitive technique, can be directly used for the early diagnosis of diabetic peripheral neuropathy. This technique mainly reflects autonomic nerve dysfunction by measuring changes in sweat electrolyte concentration (especially the ratio of sodium ions to chloride ions) [38].

Urea: Increased reabsorption of water in sweat glands leads to increased urea concentration. The transport of urea through sweat glands is based on passive diffusion, directly from blood to sweat, with the concentration of urea in sweat being 22.2 mmol/L, which is 3.6 times greater than that in serum [62]. It has been proven that an increase in serum urea (blood urea nitrogen) is strongly correlated with the incidence of DM [63]. Diabetic nephropathy occurs in 40% of patients with DM, accounting for 30–50% of end-stage renal disease, and in uremia, urea is excreted through sweat, manifesting as frost on the skin [64]. Therefore, Sudha S. et al. suggested that the increase in urea in the sweat of patients with DM may be an early sign of diabetic nephropathy [64].

Uric acid: Compared with that of urea, the content of uric acid is much lower, with the concentration of uric acid in sweat being approximately 24.5 μmol/L, which is only 6.3% of the uric acid concentration in serum. Papanas reported that the blood uric acid (SUA) level is greater in T2DM and peripheral neuropathy patients, suggesting that, in T2DM patients with impaired sweat secretion, the level of SUA increases, and it is believed that the level of SUA increases continuously with the progression of sweat gland dysfunction in both groups and the entire patient population [65].

Hormones: Studies have shown that blood glucose levels in T2DM are directly associated with cortisol levels [66]. Disruption of the circadian rhythm influenced by cortisol may result from insufficient insulin secretion, leading to increased fasting and postprandial plasma glucose concentrations [49]. This dysregulation may result from insufficient insulin secretion and consequently leads to elevated fasting and postprandial plasma glucose concentrations [66]. Although insulin, as a large molecule, is difficult to diffuse into sweat, and its concentration in body fluids, including blood and ISF, is extremely low, it remains a major challenge for future sensors [67]. Moreover, sex hormones are also considered to have anti-diabetic effects, a notion supported by the observed differences in T2DM incidence between males and females [68].

#### Lipid metabolism abnormalities

##### Obesity

Obesity is a chronic condition characterized by weight gain that affects more than 890 million adults worldwide. It is believed to result from a combination of environmental and genetic factors and is closely associated with various chronic diseases. Studies have shown that 61% of individuals with DM also exhibit obesity [69]. Obesity can affect sebaceous glands and sweat glands, leading to changes in the circulatory and lymphatic systems. People with obesity often experience excessive sweating due to larger skin folds, thicker subcutaneous fat, increased friction, and moisture, which can cause skin maceration and inflammation, leading to excessive bacterial growth. This makes them more prone to chronic conditions such as body odor (axillary odor) [70], [71]. Additionally, research has shown that pyogenic hidradenitis is closely linked to obesity. Furthermore, obesity is associated with elevated uric acid levels, which can increase the risk of gout [5], [72].Moreover, hormone levels also play an important role in obesity. Neuropeptide Y (NPY), a hormone widely present in both the central and peripheral nervous systems and involved in maintaining homeostasis, is considered closely associated with obesity [73]. Estrogen also plays a major role in the development of obesity [74], [75]. Moreover, apart from acting as an external factor that impacts the cortisol levels in hair, sweat can experience a volumetric increase as a consequence of obesity [76]. This, in turn, gives rise to higher cortisol concentrations in hair.

#### Amino acid and protein metabolism abnormalities

##### Hyperuricemia/gout

Hyperuricemia is a chronic metabolic disorder caused by acquired factors and rare genetic factors that lead to impaired purine metabolism. The only symptom is elevated blood uric acid levels, and the condition can be diagnosed when the serum uric acid (UA) concentration exceeds 7.0 mg/dl [77], [78]. Studies suggest that hyperuricemia is closely associated with the occurrence and severity of metabolic syndrome, cardiovascular diseases, and chronic kidney disease [79].

Approximately 5%-15% of individuals with hyperuricemia may further develop gout. The primary pathological feature of gout is the formation of uric acid crystals in vitro when blood UA exceeds 408 μmol/L under the combined influence of physiological pH and temperature, leading to chronic deposition and gout [80]. Changes in blood UA concentration are also considered important diagnostic indicators for gout and hyperuricemia. There is a strong positive correlation between UA concentrations in blood and sweat. Under normal conditions, the UA concentration in sweat is approximately 10% of that in blood. Therefore, sweat UA is also considered a typical biomarker for hyperuricemia and gout and is used for routine monitoring and diagnosis [81], [82].

#### Endocrine and metabolic disorders

##### Hyperthyroidism/hypothyroidism

Hyperthyroidism is considered a disease caused by the overproduction and secretion of thyroid hormones (THs), with Graves' disease being the most common cause of hyperthyroidism, manifesting as weight loss, tachycardia, heart rhythm abnormalities, anxiety, and sweating, among other symptoms [83]. Hypothyroidism, on the other hand, is caused by insufficient secretion of TH, manifesting as cold intolerance, fatigue, and bradycardia [84]. Diagnosis is generally made by examining blood levels of thyroid-stimulating hormone (TSH), triiodothyronine (T3), and thyroxine (T4), along with radioactive iodine thyroid scans and thyroid echo imaging [84]. Thyroid dysfunction has a certain impact on sweating, including excessive sweating in hyperthyroidism patients and reduced sweating in hypothyroidism patients.

Ions: Paroxysmal sweating is a typical characteristic of hyperthyroidism. Gibinski et al. reported that the average sodium concentration in the sweat of hyperthyroid patients is lower than that in the sweat of normal patients and that the chloride ion concentration is also lower, whereas the potassium ion concentration tends to significantly increase, which can be inhibited by beta-adrenergic blockers [85], [86]. Sweat glands also significantly differ between the hypothyroidism group and the normal group [87]. Hypothyroidism was once considered one of the important reasons for the increase in chloride and sodium ions in sweat [88], [89]. Many studies have shown that hypothyroid patients have significantly increased electrolyte concentrations, but Karagüzel et al., after observing 25 hypothyroid patients, believe that this statement is highly problematic [90].

Uric acid: Patients with hyperthyroidism have a significant increase in uric acid, and the kidneys also significantly increase uric acid excretion, leading to an increased uric acid clearance rate. In contrast, hypothyroidism patients exhibit a significant decrease in serum uric acid levels [91].

Hormones: Abnormal secretion of thyroid hormones is the primary cause of hyperthyroidism and hypothyroidism [49]. Although thyroid hormones are rarely secreted into sweat via sweat glands, patients with hyperthyroidism often exhibit increased sweating, which may result from enhanced oxidative metabolism or heightened activity of the sympathetic-adrenal system [92]. In addition, thyroid dysfunction can lead to changes in sex hormone levels, which can result in ovulation issues and erectile dysfunction [93].

Therefore, more valuable clinical data is needed to prove that there is a significant relationship between thyroid function and the composition of sweat.

#### Other

##### Uremia

Uremia refers to a complex systemic metabolic disorder and abnormal signaling events related to the dysfunction of various organs in the body and is caused by chronic kidney disease (CKD) [94]. Patients with uremia have a lower ratio of coil volume to duct length in their sweat glands, and they have fewer exocrine sweat glands than do normal individuals, with an average sweat output that is lower [37], [95].

Urea: Urea is eliminated via sweat glands, with its concentration in sweat typically 3–4 times higher than in serum [37]. The main reason for the increased urea concentration in the sweat of patients with uremia is an increase in the expression of the urea transport proteins UTA1 and UTB1 and a decrease in the expression of AQP5, leading to excessive release of urea and a reduction in urea excretion by the kidneys [96]. As a result, a relative increase in urea in both serum and sweat is observed. The sweat urea concentration in patients with uremia can reach 5.5–50 times that of serum, and the skin manifestations include urea frost, which is considered a significant indicator of uremia and a harbinger of renal failure [37], [62], [64], [97].

CMPF: 3-Carboxy-4-methyl-5-propyl-2-furanpropionic acid (CMPF) is considered a marker for renal dysfunction. Compared with normal individuals, patients with uremia have half the concentration of CMPF in their hair, which is believed to be due to reduced sweating [98].

Ions: Patients with uremia have significantly increased levels of Ga2+, Mg2+, and phosphate, which are inversely proportional to the rate of sweating and seem to be closely related to the increased secretion of parathyroid hormone [99], [100]. The increased content may be an important pathological factor in calcium-related pruritus syndrome associated with uremia. The potassium ion concentration in normal sweat is similar to or slightly higher than that in serum. In dialysis patients, hyperkalemia often leads to a relatively high mortality rate. In uremic patients, the potassium ioVn concentration in sweat is four times higher than that in plasma [101]. This may be because severe hyperkalemia exceeds the reabsorption capacity of the sweat gland ducts, so that potassium ions cannot be completely reabsorbed, and a large amount of potassium ions are excreted into the sweat in the form of net secretion.

Metabolism-related diseases are closely related to genetic factors, environmental factors, and metabolic disorders, and the components of sweat, both inorganic and organic, are important metabolic products within the body [102]. In addition, endocrine and metabolic diseases can cause changes in the function of sweat glands and the composition of sweat, increasing the value of sweat testing for the diagnosis of endocrine and metabolic diseases.

### Disease of respiratory system

#### Cystic fibrosis (CF)

CF is a congenital disease with familial autosomal recessive inheritance and is also considered a disease of the exocrine glands [103]. The level of chloride in sweat is usually less than 30 mmol/L and less than 40 mmol/L in the sweat of elderly individuals and infants [2].In CF patients, dysfunctional or absent CFTR impairs Cl⁻ reabsorption, leading to markedly elevated sweat Cl⁻ concentrations (>60 mmol/L), which remains the gold-standard diagnostic criterion [34]. CF can activate the Cl- channel, and mutations in CF disrupt chloride levels [40]. In CF patients, although the levels of Na+ and Cl- in sweat are high, the levels of HCO3- and pH remain unchanged [104]. Sweat Na⁺ is secondarily elevated due to reduced paracellular Na⁺ reabsorption in the absence of luminal electronegativity generated by Cl⁻ transport [2]. In addition, the increased concentration of Cl- in sweat in various pulmonary diseases, such as chronic obstructive pulmonary disease (COPD) and emphysema, is considered to have an important relationship with the abnormal function of CF [105], [106], [107], [108].Studies have found that patients with CF exhibit significant abnormalities in glucocorticoid metabolism, with markedly reduced cortisol metabolism [109]. However, there is currently no direct evidence showing altered cortisol levels in the sweat of CF patients.

In addition, since COPD is closely related to chronic inflammation, the serum and sweat C-reactive protein (CRP) values are higher in former smokers than in current smokers [30].

### Circulation system disease

#### Heart failure (HF)

HF is a disease caused by abnormal ventricular diastolic filling and a reduced ejection fraction during systole, and it is one of the main causes of complications and death from most diseases [110]. Hyperhidrosis is a common symptom of HF in children and adults and is also a manifestation of early HF cardiac maladjustment [111]. In some cases, fluid overload in HF may be a compensatory mechanism of the body for excessive volume load. Compensation. An overactive sympathetic nervous system increases the reabsorption of fluid in the kidneys. On the other hand, it generates signaling to the sweat glands, inducing fluid dispersion and resulting in sodium and chloride losses at the level of the glandular ducts. Finally, the physiological functions of sweat glands in HF patients also change during physical activity. More importantly, there are significant impairments in the thermoregulatory function of HF patients. Although the core body temperature and overall sweating rate are similar to those of healthy individuals during exercise or heat stress, the ability to regulate skin blood flow is significantly impaired, manifested as a weakened skin vasodilation response, leading to a reduced efficiency of heat dissipation. Therefore, even if the sweating volume is not reduced, the physiological effectiveness of sweating is limited due to circulatory disorders, further highlighting the value of sweat metabolites as a non - invasive monitoring tool [112].

Lactate: Experts and clinical guidelines recommend that cardiovascular patients with heart failure engage in appropriate aerobic exercise, that the lactate threshold (SLT) and ventilatory threshold (VT) are key components for optimizing exercise, and that there is a strong correlation between the two. Katsumata Y et al. observed 50 HF patients and reported that the power of the SLT was positively correlated with the cardiac threshold. In NYHA Class I and II HF patients, disease progression can be detected through the level of lactate in sweat, but the sensitivity is slightly poor in NYHA Class III patients. The study also points out that patients with a moderate sweating rate (approximately 0.43 mg/min/cm²) are most suitable for using this type of sensor to determine sweat lactate [113].

Ions: The main components of sweat are water and electrolytes, which are also additional pathways for Na+ and fluid clearance in HFs. The normal concentration of Na+ in sweat is 15–60 meq/L [114], [115]. The concentration of Na+ in HFs changes but is comparable to the concentration of urinary Na+ after diuretic treatment. There is evidence that excessive circulation of adrenergic electrolytes is part of the mainstream Na+ in congestive HF, and Keynocds's research also proved that the concentration of Na+ in sweat in HFs exceeded this limit [115].

Protein: CRP is closely related to inflammation and death in several disease states. Chronic systemic inflammation also increases the risk of cardiovascular events and is widely used for the detection of cardiovascular diseases [116]. Tu et al. reported that the CRP level in the sweat of patients with preserved ejection fraction (HFpEF) was significantly increased, whereas there was no increase in those with reduced ejection fraction (HFrEF). It suggests that it may have specific value in the monitoring of HFpEF. [30].

Amino acids: Amino acids are also important components of sweat. When standardized to a comprehensive or complete amino acid percentage, the content of amino acids, especially serine and glycine, in HF patients increases, which seems to be related to the process of glycolysis [117].

Hormones: Patients with heart failure exhibit significant changes in the diurnal rhythm of cortisol and have markedly lower overall cortisol levels compared to healthy individuals [118]. Serum cortisol is regarded as an independent predictor of chronic heart failure events [119]. Therefore, monitoring changes in sweat cortisol concentration, which allows for continuous assessment, may be a promising approach for tracking heart failure events. Moreover, testosterone deficiency occurs in 26–37% of male heart failure patients, and is therefore considered an independent predictor of heart failure prognosis [120].

### Autoimmune diseases

#### Behcet's disease(BD)

BD is a rare chronic multisystem inflammatory disease, the pathogenesis of which involves multiple factors, such as infection, genetics, epigenetics, and immunology [121]. Because patients often have a special and unpleasant odor when seeking medical attention, Cui and others believe that it may be closely related to the secretion of sweat metabolites. A total of 175 metabolites were identified in the sweat of BD patients. Like in blood and urine, a significant decrease in L-citrulline, which may be an important pathogenic metabolite, can also be found in the serum, urine, and sweat of BD patients [122].

#### Vogt—Koyanagi—Harada (VKH) disease

VKH disease is an autoimmune disease that causes visual impairment [123]. Cui et al. used liquid chromatography—tandem mass spectrometry to analyze the proteome and metabolome of 60 sweat samples and reported significant differences in 116 proteins and 21 differentially expressed metabolites in VKH patients [26]. The former includes 17 proteins with increased expression and 99 proteins with decreased expression, whereas the latter includes 18 metabolites whose expression increased and 3 whose expression decreased [26].

#### Inflammatory bowel disease (IBD)

In IBD patients, inflammatory factors are important indicators for detection. When detecting TNF-an in the sweat of IBD patients, the average level of TNF-an in IBD patients is nearly 20 times greater than that in healthy individuals [124]. Changes in the levels of inflammatory indicators such as IL-1β, IL-6, and CRP are also key focuses of attention [125], [126]. Sex hormones are currently believed to influence the development of IBD through multiple mechanisms, including immune regulation and gut microbiota remodeling [127]. Therefore, monitoring sex hormone levels in sweat is theoretically highly promising for understanding the hormonal regulatory mechanisms of IBD and developing novel non-invasive monitoring approaches, but there is currently no direct evidence supporting its clinical feasibility.

### Dermatological afflictions

#### Atopic dermatitis (AD)

**Fig. 3:**
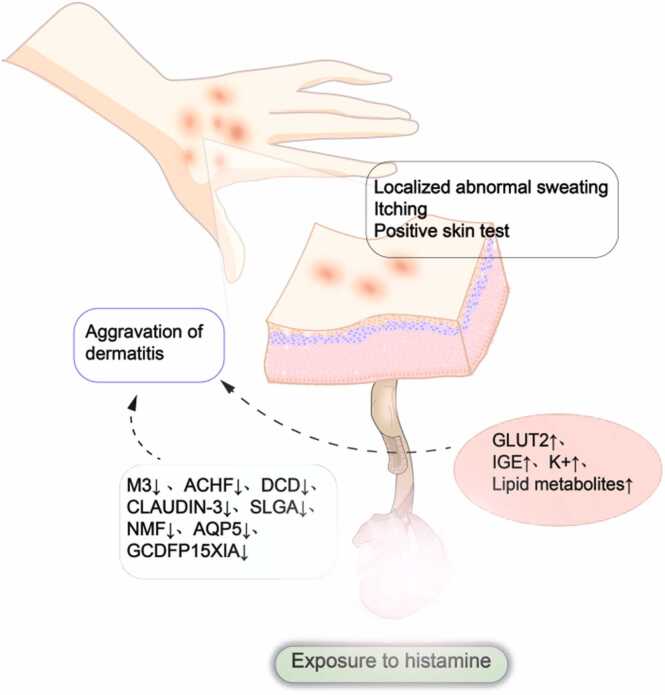
**| Sweat in dermatological afflictions.** Sweat plays a key role in dermatological afflictions, manifesting as localized sweating dysfunction and itching. Most AD and CholU patients have positive skin tests. With changes in certain components of sweat, the occurrence of dermatitis is significantly aggravated.

AD is a typical skin lesion with abnormal sweating ability, and the lesions are characteristically distributed in areas with prominent sweat glands, such as the neck, face, wrists, and antecubital and popliteal fossae [128]. Most AD patients test positive for autologous skin [9]. In the report by Williams et al., sweat was considered one of the main aggravating factors of AD across all age groups, and the study revealed varying degrees of changes in glucose, ions, proteins, amino acids, and lipids in sweat, as illustrated in Fig. 3 [129].

Glucose: Ono and others compared the concentration of glucose in the sweat of AD patients with that in non-AD patients and reported that the concentrations of glucose and the mRNA of the glucose transporter GLUT2 in the former were significantly increased, especially during the acute phase, and there was potential cavity heterotopy [130]. On the basis of sweat NMR metabolomics, glucose is considered an important marker of the severity of the disease [2].

Ions: AD patients have a relatively greater pH (5.54) on nonlesional skin than do normal individuals (5.24) [131]. The increased secretion of sweat leads to incomplete reabsorption of bicarbonate from sweat gland ducts. Bicarbonate increases the pH of sweat, and when excess sweat remains on the skin surface, it can irritate sebaceous glands and cause skin inflammation [29]. Liebke compared AD children with normal children and reported that the median concentration of NA^+^ in sweat was significantly greater in the former, whereas mild AD patients presented significant decreases in Na^+^, K^+^, lactate, urea, and PCA [29], [132].

Proteins: AD patients have a weakened innate immune response of the skin to pathogens, which may be related to a significant reduction in antimicrobial DCD peptide and secretory immunoglobulin A (sIgA) levels [28], [29]. Forrestom reported that the total IgE level in the sweat of AD patients is directly proportional to the severity of the disease, which seems to be related to the byproduct antigen MGL-1304 of Malassezia [133], [134], [135]. MGL-304 is composed of a small amount of protein in sweat but has specific allergic activity, which can induce the release of histamine in AD patients [129]. In addition, since atopic dermatitis is more likely to result in functional abnormalities of the stratum corneum (SC), studies on proteins in the SC suggest that increased levels of KLK and protease inhibitor (CEKT1) and decreased levels of gross cystic disease fluid protein 15 (GCDFP15) are closely related, and GCDFP15 is also considered a potential marker of sweating disorders in AD [136], [137]. AD patients exhibit skin malignancy, and IL-37 and claudin in sweat are considered related to this phenomenon [128]. In AD patients, the concentrations of LL-37 and DCD in sweat have been characterized and are believed to be associated with susceptibility to AD skin lesions. Additionally, compared with that in healthy skin, the expression of claudin-3 in the sweat glands of AD patients is significantly lower [128].

Amino Acids: Free amino acids in sweat may differ somewhat by sex but not significantly, and natural moisturizing factor (NMF) is considered a skin marker related to the integrity of the SC; thus, a decrease in NMF levels is observed in AD patients [25].

Lipids: The metabolism of sphingolipids, ceramides and other related lipid substances is increased in AD patients, especially in male patients, which can be up to six times greater and is also considered to have great potential for diagnosing this disease [138].

Hormones: Sex hormones play an important role in AD by modulating immune responses and affecting skin barrier function.Studies have shown that the prevalence of AD in females is significantly higher than in males after puberty, and sex hormones are therefore considered one of the key factors contributing to the gender differences and disease course fluctuations in AD [139].

#### Cholinergic urticaria (CholU)

CholU is a characteristic type of urticaria marked by pinpoint-sized wheals associated with sweating (including activities such as taking hot showers, exercising, or emotional stress) along with itching and/or tingling [140]. CholU is a characteristic type of urticaria that is induced mainly by sweat, and the wheals it exhibits are believed to be mediated by acetylcholine. In Bito T's study, 64.7% of CholU patients had positive skin tests [12], [141]. Additionally, 66% of CholU patients show histamine release in response to semipurified sweat antigens. Like in AD patients, MGL-1304 in sweat is also an important allergen [133], [141]. Wang Y reported that CholU patients also exhibit a deficiency in sweat gland acetylcholine receptor M3 and acetylcholinesterase (ACHE), which may be closely related to impaired sweating in CholU patients [142].

Additionally, the histamine concentration in the sweat of CholU patients is much greater than the histamine concentration in the plasma of healthy subjects. The high concentration of histamine in sweat may be due to the infiltration of histamine released from skin mast cells in response to sweat antigens near the sweat glands and ducts [140]. Therefore, histamine can be used as an important marker for monitoring CholU. A recent study revealed that local sweating in CholU patients is partially and severely reduced, but the total sweat volume remains unchanged. Compared with healthy controls, CholU patients exhibit tight junction (TJ) dysfunction in sweat glands, which leads to increased concentrations of K⁺ and Ca²⁺ in sweat [143].

#### Hidradenitis suppurativa (HS)

HS is a disease of the apocrine sweat glands [144]. Studies on HS have revealed that the expression of genes related to sweat gland function (WIFI, AQP5, FOXA1, etc.) is significantly reduced, whereas the expression of genes related to immunity (IL-37, Hbd3, MIF, etc.) is increased, indicating a clear inflammation-driven phenomenon [145], [146].

### Cancer

In 2020, there were 19.3 million new cancer cases, which is expected to increase to 27.5 million cases by 2024, making it one of the leading causes of death. Currently, methods such as gas chromatography—mass spectrometry are used to analyze organic compounds in sweat to evaluate biomarkers for cancer [2], [147].

Proteins: The proteinase-induced factor (PIF) derived from the N-terminus of the DCD protein secreted by eccrine glands is believed to be a peptide processed by tumor cells and is thought to play multiple roles in cancer cachexia [42].

Uric acid: Hyperuricemia is closely linked to the incidence and metastatic risk of cancer. Given the strong correlation between sweat and blood uric acid levels, as demonstrated by recent wearable biosensor studies, non-invasive monitoring of uric acid dynamics in sweat offers a promising approach for tracking systemic metabolic status and disease progression in cancer patients [81].

Carbohydrates: Calderón-Santiago and others analyzed the sweat metabolomics of lung cancer and reported that lung cancer has a relatively high PAVC value and that the most specific chemicals are nonanedioic acid and tetrahexose, which are related to amylases, with significant changes in cancer tissues [148].

Hormones: Cortisol is considered one of the products of chronic stress [149]. Chronic stress can lead to hyperactivity of the hypothalamic-pituitary-adrenal (HPA) axis, resulting in persistently elevated cortisol levels, which in turn creates an immunosuppressive microenvironment that promotes tumor drug resistance and thereby influences cancer progression [150], [151], [152]. Current cortisol research related to cancer primarily relies on hair, serum, and saliva [76]. Cortisol is the most abundant hormone in sweat compared to other hormones, making sweat-based cortisol measurement theoretically feasible as well [153]. Moreover, sex hormones and their receptors also play a critical role in cancer management. It can be used to assess the risk, treatment response, and prognosis of hormone-related cancers such as breast, prostate, and endometrial cancer, providing strong support for personalized cancer screening and health management [154], [155], [156]. However, like cortisol, it still requires specific clinical data for validation.

## Challenge

Although sweat provides a non-invasive and easily obtainable biofluid, its analytical reliability is affected by several physiological and methodological factors. The composition of sweat varies across collection sites (forearm, back, forehead, etc.) [64] and depends strongly on sweat rate, which influences dilution and ion concentration. Environmental temperature, hydration status, and emotional stress can further modify secretion. Additionally, skin contamination and evaporation introduce variability [44].Although a moderate correlation exists between analytes in sweat and those in plasma, suggesting potential semi-quantitative diagnostic utility, sweat analysis has not yet achieved the accuracy required for definitive diagnosis and must be combined with other diagnostic methods.

To improve reproducibility, standardized procedures—controlled sweat rate stimulation, site-specific collection patches, and normalization to sweat rate—are recommended. Future work should also validate intra-individual stability and inter-individual variability through repeated sampling.

## Conclusions

Analysis of sweat composition plays a crucial role in medical diagnosis and disease monitoring. Changes in sweat components not only serve as potential biomarkers but also reflect underlying pathophysiological disturbances，particularly those involving systemic homeostasis, glandular transport function, and inflammatory or neuroendocrine regulation. For example, alterations in glucose, ions, proteins, amino acids, and lipids have been linked to a range of conditions, including metabolic, endocrine, and nonmetabolic diseases. These changes provide insights into disease mechanisms and support early detection, accurate diagnosis, and personalized management.

1) Diagnostic Value: Variations in sweat composition can serve as potential biomarkers for the diagnosis and monitoring of a wide range of diseases. For example, in metabolic disorders such as DM, changes in sweat levels of glucose, lactate, chloride, urea, and uric acid have demonstrated diagnostic relevance. Similarly, the elevated sodium chloride concentration in sweat is a well-established diagnostic hallmark of cystic fibrosis. Beyond these established examples, emerging evidence suggests that organic compounds, cytokines, and metabolites in sweat may offer non-invasive indicators for cancers, neuropsychiatric disorders, and autoimmune conditions.
2) Reflection of Disease Mechanisms: Alterations in sweat composition often reflect underlying pathophysiological processes, particularly those involving inflammation. In hidradenitis suppurativa, for instance, an inflammation-driven phenotype is associated with downregulation of sweat gland functional genes and upregulation of immune-related genes. In cardiovascular diseases such as heart failure, elevated sweat CRP levels correlate with chronic systemic inflammation, highlighting the role of inflammatory pathways in disease progression. Furthermore, sweat composition mirrors specific disease mechanisms: hypoglycemia in DM triggers neurohormonal responses that disrupt sweat gland function; thyroid dysfunction (hyper- or hypothyroidism) alters electrolyte and uric acid homeostasis in sweat; and in autoimmune conditions like Behçet’s syndrome and VKH syndrome, immune-mediated changes are reflected in sweat profiles, offering insights into disease etiology
3) Disease monitoring and prognosis assessment: Sweat analysis also holds promise for longitudinal monitoring and prognostic evaluation. In atopic dermatitis, sweat concentrations of glucose and proteins correlate with clinical severity, enabling objective disease tracking. In heart failure patients, dynamic changes in sweat lactate and CRP levels reflect disease trajectory and systemic stress. Likewise, monitoring uric acid fluctuations in sweat may provide real-time insights into tumor metabolism and treatment response in cancer patients. Together, these applications illustrate how sweat-based metrics can support personalized and proactive disease management.

Physiological constraints must be acknowledged: Sweat is not a simple ultrafiltrate of plasma; it is a rate-limited, selectively modified fluid shaped by eccrine gland biology. Biomarker concentrations are influenced by sweat rate, local gland density, circadian rhythm, and interindividual variation in transporter expression. Without accounting for these factors—and without mechanistic validation—the clinical translation of sweat biomarkers remains limited. Future work should prioritize linking molecular changes in sweat to defined physiological pathways, ensuring robustness beyond disease labels.

## CRediT authorship contribution statement

**Changqi Shi:** Writing – review & editing, Writing – original draft. **Ziyi Chen:** Writing – review & editing, Writing – original draft. **LuLu Zhang:** Writing – review & editing, Conceptualization. **Xiaomeng Zhang:** Visualization, Conceptualization. **Xiaoyu Liang:** Validation, Conceptualization. **Lan Yan:** Methodology, Investigation. **Xiaopeng Hao:** Supervision, Software. **Guoju Dong:** Validation. **Cheng Lu:** Visualization, Validation. **Luqi Huang:** Visualization.

## Ethics declaration

Not applicable.

## Funding Declaration

Scientific and Technological Innovation Project of 10.13039/501100005892China Academy of Chinese Medical Sciences (CI2023C010YL).

## Conflict of Interest

The author has no conflicts of interest to disclose. Fig. 2 and the graphical abstract were created with Figure Draw (www.figdraw.com).

## Declaration of Competing Interest

The authors declare that they have no known competing financial interests or personal relationships that could have appeared to influence the work reported in this paper.

## Data Availability

No new data were created or analyzed in this study. Data sharing is not applicable.
